# The Application of a Serious Game Framework to Design and Develop an Exergame for Patients With Heart Failure

**DOI:** 10.2196/50063

**Published:** 2024-08-07

**Authors:** Aseel Berglund, Tiny Jaarsma, Helena Orädd, Johan Fallström, Anna Strömberg, Leonie Klompstra, Erik Berglund

**Affiliations:** 1 Department of Computer and Information Science Linköping University Linköping Sweden; 2 Department of Health, Medicine, and Caring Sciences Linköping University Linköping Sweden; 3 Department of Cardiology Linköping University Linköping Sweden

**Keywords:** mobile health apps, physical activity, exergames, player-centered design, heart failure, human-computer interaction, mobile phone

## Abstract

Reducing inactivity in patients with chronic disease is vital since it can decrease the risk of disease progression and mortality. Exergames are an innovative approach to becoming more physically active and positively affecting physical health outcomes. Serious games are designed for purposes beyond entertainment and exergames are serious games for physical activity. However, current commercial exergames might not optimally meet the needs of patients with special needs. Developing tailored exergames is challenging and requires an appropriate process. The primary goal of this viewpoint is to describe significant lessons learned from designing and developing an exergame for patients with chronic heart failure using the player-centered, iterative, interdisciplinary, and integrated (P-III) framework for serious games. Four of the framework’s pillars were used in the design and development of a mobile exergame: player-centered design, iterative development of the game, interdisciplinary teamwork, and integration of play and serious content. The mobile exergame was developed iteratively in 7 iterations by an interdisciplinary team involving users and stakeholders in all iterations. Stakeholders played various roles during the development process, making the team stay focused on the needs of the patients and creating an exergame that catered to these needs. Evaluations were conducted during each iteration by both the team and users or patients according to the player-centered design pillar. Since the exergame was created for a smartphone, the assessments were conducted both on the development computer and on the intended platforms. This required continuous deployment of the exergame to the platforms and smartphones that support augmented reality. Our findings show that the serious game P-III framework needs to be modified in order to be used for the design and development of exergames. In this viewpoint, we propose an updated version of the P-III framework for exergame development including (1) a separate and thorough design of the physical activity and physical interaction, and (2) early and continuous deployment of the exergame on the intended platform to enable evaluations and everyday life testing.

## Introduction

A physically inactive lifestyle is increasingly common in the Western world [1]. An inactive lifestyle is characterized by low levels of moderate to vigorous intense physical activity and high levels of sedentary behavior (ie, sitting or lying down) [2,3]. A meta-analysis has shown that the negative health effects of an inactive lifestyle have the highest impact on individuals who have both a low amount of exercise and a high amount of sedentary behavior [4,5]. Patients with a cardiac disease typically report low physical activity levels, which increases the risk of disease progression and mortality [6-8]. Physical activity is the cornerstone of cardiac rehabilitation, but even after cardiac rehabilitation, most patients return to an inactive lifestyle within months [9,10]. These high levels of physical inactivity are found across multiple cardiac diagnoses [11,12]. Heart failure is one of the cardiac conditions that are lifelong and can be described as a condition in which the heart muscle cannot pump enough blood to meet the body’s needs for blood and oxygen. Basically, the heart cannot keep up with its workload, meaning that the patient gets tired much more quickly. Contrary to what many people believe, physical activity is considered beneficial to patients with heart failure, though it needs to be adapted to the severity of symptoms and the baseline condition of the patient [13].

A novel approach to improve physical activity is the use of exergames, namely video games that require physical activity and movement to play. Exergames are serious games for health [14] that can increase motivation to be more active [15] and have been found to improve a patient’s physical function, balance, physical activity, exercise capacity, and energy expenditure [16-21]. However, commercial exergames with ‘‘one size fits all’’ approaches do not meet the needs of older people in general [22,23] and patients with heart failure in particular [24]. A randomized controlled trial study using an “off the shelf” commercial product was not effective in improving submaximal exercise capacity in patients with heart failure [25]. For patients with heart failure, achieving the desired effects of playing exergames requires a tailored design that addresses their specific needs.

A challenge for the development of serious games is that normal game development is costly, for example, the development budget for high-quality entertainment games is at least US $1 million, while many serious game projects need to work within a limited budget, which can result in games with low quality or a significant discrepancy between user expectations and the quality of gameplay [26]. Producing a successful serious game requires involving both professional game developers and users in the design process [26]. The player-centered, iterative, interdisciplinary, and integrated (P-III) framework offers a method for designing and developing serious games [27].

The main goal of this viewpoint is to describe significant lessons learned from designing and developing an exergame for patients with chronic heart failure using the P-III serious game framework and propose an updated version of the framework adapted for developing exergames for health.

## The Serious Game P-III Framework

### Overview

Developing serious games for health is challenging since it involves combining serious and entertaining goals, involves large-scale software development with a multidisciplinary team, and the users are not identical [28]. The P-III framework mitigates these challenges by providing a player-centered, iterative, and integrated approach to designing and developing serious games for health with a multidisciplinary team [27]. The framework was established through research projects where it was formed, designed, verified, and refined [29]. The framework consists of 4 conceptual pillars.

### Pillar 1: Player-Centered Design

To create games that meet the needs of the target group, players are involved in the entire process of game design and development, rather than involving them only in the usability testing phase [27,30,31]. The process is based on the human-centered design process [32-34].

### Pillar 2: Iterative Development of the Game

**Figure 1:**
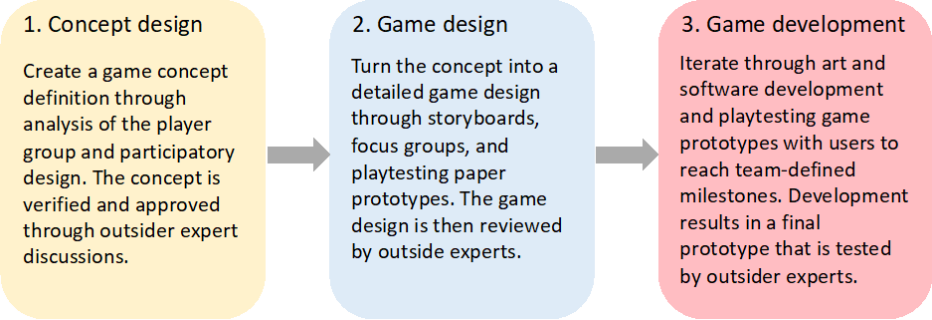
The three phases of the iterative design and development of the serious game according to the P-III: player-centered, iterative, interdisciplinary, and integrated framework [27].

The game is designed and developed iteratively and with three phases (Figure 1) [27] as follows: (1) concept design to understand the player group and the problem domain, (2) game design, where the game concept is transformed into a detailed game, and (3) game development, where a prototype is built and tested with users.

### Pillar 3: Interdisciplinary Teamwork

In addition to software engineers, visual artists, project managers, and game designers, serious game production requires the expertise of social scientists and other subject matter specialists [27].

### Pillar 4: Integration of Play and the Serious Content

The serious content is integrated with the play as closely as possible [27]. The development is based on theory-driven and evidence-based frameworks [35] and should focus on integrating serious content [36].

## Case: Developing an Exergame Using the Serious Game P-III Framework

### Overview

This viewpoint summarizes the design and development process of a mobile exergame Heart Farming [37] using the P-III framework to share the phases in the process so other health app developers and scientists might reuse or extend the process.

**Figure 2:**
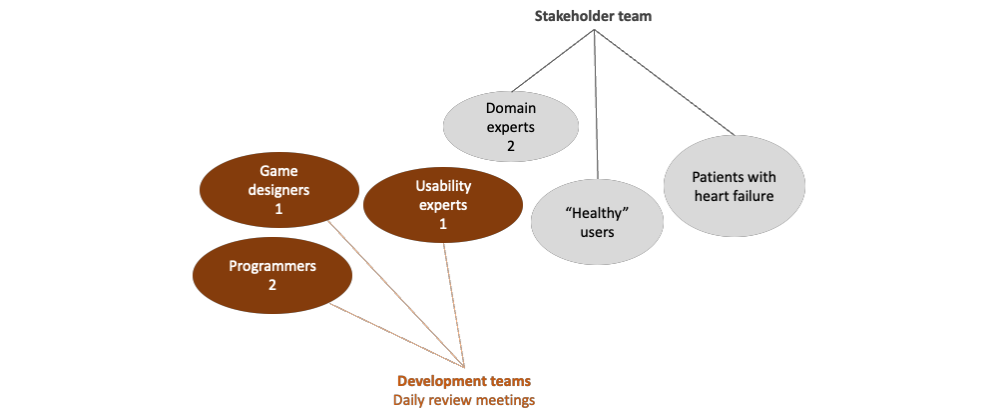
Heart Farming interdisciplinary team.

**Figure 3:**
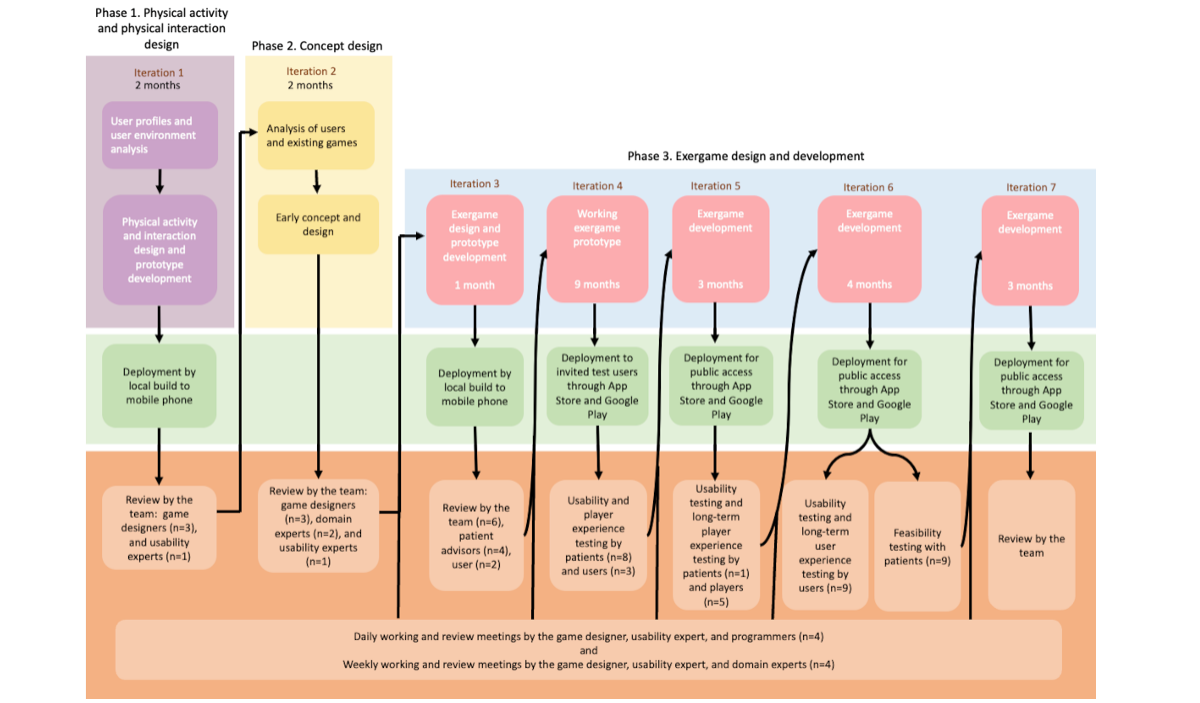
The Heart Farming exergame design and development process.

The exergame was developed by an interdisciplinary team consisting of a development team (1 game designer, 1 usability expert, and 2 programmers), stakeholders (as domain experts, patients with cardiac disease, and users who were not patients with heart failure called “healthy” users), and users (patients with cardiac disease and users with no cardiac disease; Figure 2). The exergame was developed through 7 iterations until it was safe, efficient, and entertaining (Figure 3). All iterations included deployment and evaluations, so the exergame was continually tested by the team. All iterations included deployment and evaluations, so the exergame was continually tested by the team. In each iteration, new patients and “healthy” users (who are not patients with heart failure and do not know if they have any other diseases) participated in testing and evaluations. In this section, we summarize the iterations.

### Iteration 1

#### Iteration Goal

The iteration goal was to design the physical interaction and define the intended platform for the exergame and desired physical activity.

#### Outcome

Previous studies focused on patients with heart failure have shown a relationship between physical activity and health outcomes, where mortality and hospitalization risk decrease with even just 10 minutes of additional physical activity per day [38]. A previous study showed that outdoor mobile exergames increase older adults’ engagement, satisfaction, and interest in physical activity [39]. Furthermore, previous research showed that the quality of life of patients with heart failure can be improved by using mobile phones as health interventions to stimulate physical activities and exercise monitoring [40]. It thus was determined that the end users (patients with heart failure who are usually older people) needed to increase their physical activity by walking an additional 10 minutes per day, both indoors and outdoors. Furthermore, the ability for end users to play exergames with others has previously been identified as important for the intended end users [24]. Therefore, an exergame running on a smartphone was considered to be a suitable solution for the purpose. Indoor walking and detecting activities, such as squats, turned the focus to mobile augmented reality which allows for tracking changes in position in a 3D space without the need of, for example, using the accelerometer to detect specific motions to see when the player is walking or doing a squat.

The integration of play with this content was focused on following game design guidelines [41,42], exergame design guidelines for older people [24,43], game design principles in everyday fitness apps [44], and the patient’s perspectives [45]. The guidelines included setting a suitable level of difficulty for the physical conditions [43,46], avoiding the provision of feedback about not achieving a goal or not performing an activity [47], and developing a user interface that would be usable by older players [43,46] by avoiding small objects and developing an easy-to-use interface [43]. These guidelines were identified early in the design process.

The players’ physical condition has to be taken into account and an appropriate level of difficulty needs to be considered [43,46]. Furthermore, the risk of injuries needs to be minimized, the older adults’ extended reaction times and overall movement durations need to be considered, and the increased risk of falls has to be acknowledged [48]. Therefore, players should be able to play the exergame without the need to look at the screen while walking and the reward system should not lead to overexertion. Unlike multitouch for smartphones or mouse and keyboard for desktop apps, the physical interaction design of an exergame needs to be considered as a separate design challenge. A digital prototype was developed using mobile augmented reality. The exergame concept was to move around in a 3D map by turning the smartphone and walking in a particular direction. This was intended to be used for a gardening app, wherein the user would manage plants in a simulated indoor garden. Testing with usability experts and domain experts showed that the interaction was too complicated and potentially unsafe for the patients. Since the risk of injury had to be minimized [48] and the user interface needed to be easy to use so the player could focus on the physical activity without unnecessary complexity [47] although the experience was immersive and engaging, this design was abandoned. Thus, the decision was made to use mobile augmented reality only to enable the smartphone to track the user’s movement in meters while walking, without the need to interact with the exergame on the screen while walking.

### Iteration 2

#### Iteration Goal

The iteration goal was to define the exergame concept.

#### Outcome

Since it is recommended to use a topic tailored to the interests of older adults [43,46,47,49], farming as a game activity was chosen because (1) tending to a farm is a known concept to the user group, (2) farming is a common game theme with examples of games with large audiences who play for long periods of time, (3) farming has many possible engagement levels, from optimizing farming activities (eg, so and harvest) to take care of the farm, which can provide suitable gameplay, and (4) the graphics for the exergame could be acquired through public domain resources. Then, 2 popular farming mobile games, Hay Day and Farmville 2: Country Escape, were downloaded and played by the usability expert in the development team for inspiration.

**Figure 4:**
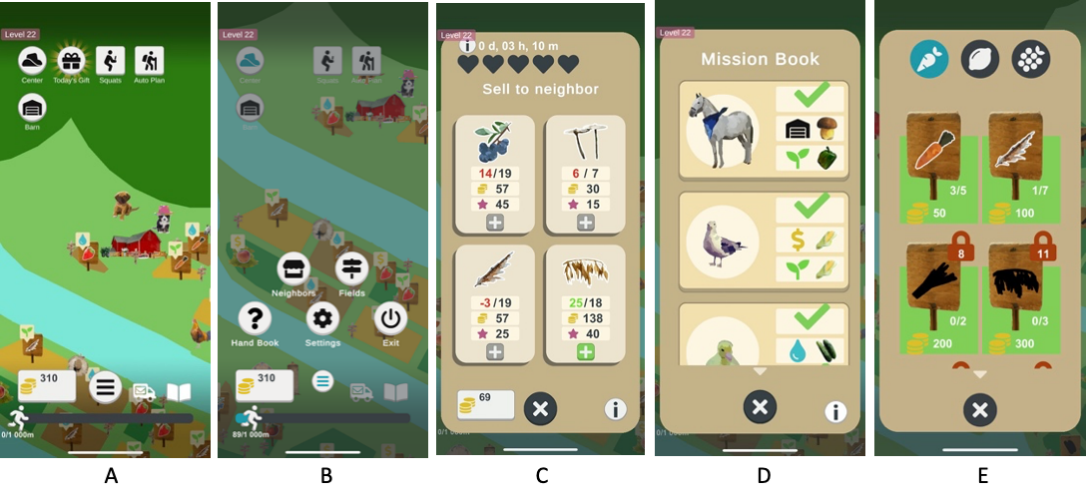
The final version of the Heart Farming exergame showcasing its interface and key features: (A) the main scene with buttons and information, (B) the main menu, (C) sell orders, (D) a mission book containing different missions, and (E) all available crops on the farm displayed and accessed by a field menu.

The exergame concept is taking care of a farm and expanding it by sowing, watering, and harvesting crops (Figure 4). To do activities on the farm (eg, watering), patients must walk in the physical world, indoors or outdoors. Each activity is rewarded with experience points.

### Iteration 3

#### Iteration Goal

The iteration goal was to develop the first digital prototype of the exergame concept.

**Figure 5:**
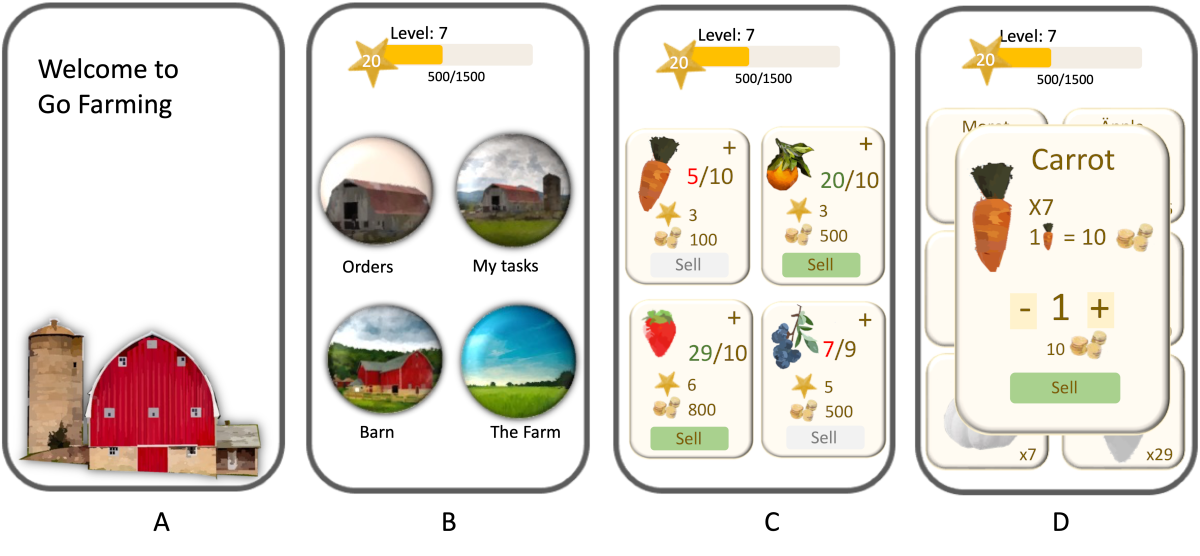
Low-fidelity prototypes were developed by the game designer and usability expert to illustrate and examine concepts and designs: (A) start menu, (B) main scene, (C) sell orders, and (D) sell card.

The exergame was developed and reviewed once a week by the stakeholder team. The development team presents updates and the stakeholder team tests them. Low-fidelity prototypes (Figure 5) and high-fidelity prototypes were developed to illustrate the design details and interaction.

#### Outcome

The concept is transformed into a first prototype by creating a game loop and adding gaming elements.

### Iteration 4

#### Iteration Goal

The iteration goal was to develop a working prototype and evaluate the prototype on the intended platform by the team and together with users to (1) verify the ability to capture the physical activity, (2) evaluate the feasibility of tracking the physical interaction, and (3) evaluate the player experience at an early stage.

A working prototype was developed in the Unity game engine and continually tested by both the interdisciplinary team and 3 “healthy” users. When the prototype was stable, a usability and user experience test was conducted by 8 patients in the Swedish Heart and Lung Association. The patients had not seen the exergame before the test. The patients were invited to a workshop that started by introducing the purpose of the exergame. Each patient was then asked to play the exergame and was observed while they used the exergame. Following testing, an unstructured interview was carried out with each patient to investigate the patients’ experience using and interacting with the exergame.

#### Outcome

The patients had difficulties understanding how to play the exergame. Therefore, the need for an instructional tutorial that explains how to play and interact with the exergame was identified.

### Iteration 5

#### Iteration Goal

The iteration goal was exergame development and usability testing.

The prototype was further developed and released via the Apple App Store and Google Play to be downloaded on smartphones and evaluated by a patient and 5 other users, none of them had seen the exergame before the test. One user borrowed a smartphone since the exergame did not work on the user’s smartphone. The evaluation started with a usability testing in which the participants ran through the tutorial first. The think-aloud protocol [50] was used while the participants were interacting with the exergame and being observed by an instructor. When the participants felt they had understood the exergame they were instructed to use the exergame for 1 week and achieve the goal of walking 10 minutes every day. The participants received a telephone follow-up after 2 days and then after another 2 days. During the follow-ups, the participants were asked what they thought about the exergame. A final follow-up unstructured interview was conducted at the end of the test period to collect data about their experience of the exergame.

#### Outcome

Usability problems were identified.

### Iteration 6

#### Iteration Goal

The iteration goal was to fix usability problems from the previous iteration and conduct feasibility testing with patients with heart failure and usability testing with “healthy” users in parallel.

First, a feasibility test with 9 patients with heart failure was conducted. The patients had not seen the exergame prior to the test. The test started with an introduction to the exergame. The patients went through the tutorial of the exergame together with the instructor, who explained the exergame in more detail. Patients were encouraged to seek clarification or ask questions in case any aspect was unclear or if they found difficulty in understanding any information. The patients were then instructed to enhance their daily walking activities according to their physical capabilities by playing the exergame for 1 month. A total of 6 patients downloaded the exergame onto their smartphones while the rest borrowed smartphones from the team. In total, 6 follow-up telephone calls were made to the participants (on days 2 and 4 and after 1, 2, 3, and 4 weeks).

Second, a usability test and a long-term player experience test were conducted with 9 users who had not seen the exergame before the test. A total of 8 participants downloaded the exergame onto their smartphones while 1 borrowed a smartphone from the team. The participants were instructed to think aloud [50] while testing the exergame and they were observed by the instructor. The participants started the test by going through the tutorial by themselves. After the usability testing, the participants were asked to use the exergame for a week and to achieve their daily goal every day, walking 10 minutes every day. At the end of the test period, a semistructured interview was conducted with each participant asking the following questions about the exergame:

- Describe your expectations for the game before you started using it?
- Do you feel that the exergame met your expectations? If Yes or No, what do you think is the reason?
- Describe a typical day when you used the exergame?
- How do you feel that exergame has affected your life situation?
- What did you like most about the game? Why?
- What has been challenging in the game?
- How have you experienced:
  the introduction to the game?
  various rewards, such as the trophy you get when you finish the daily goal or the animals placed on the farm?
  play together by, for example, to send things to others?
  the sounds from the exergame?
  the graphics?
- How do you think the game can change?
- Would you like to continue using the game? Why?
- Is there anything you would like to add or ask about?

#### Outcome

Minor usability problems in the user interface were identified.

### Iteration 7

#### Iteration Goal

The iteration goal was to fix the minor usability problems in the user interface and finalize the exergame without adding any new features.

#### Outcome

The exergame is released at App Store and Google Play.

## Discussion

### Principal Findings

**Figure 6:**
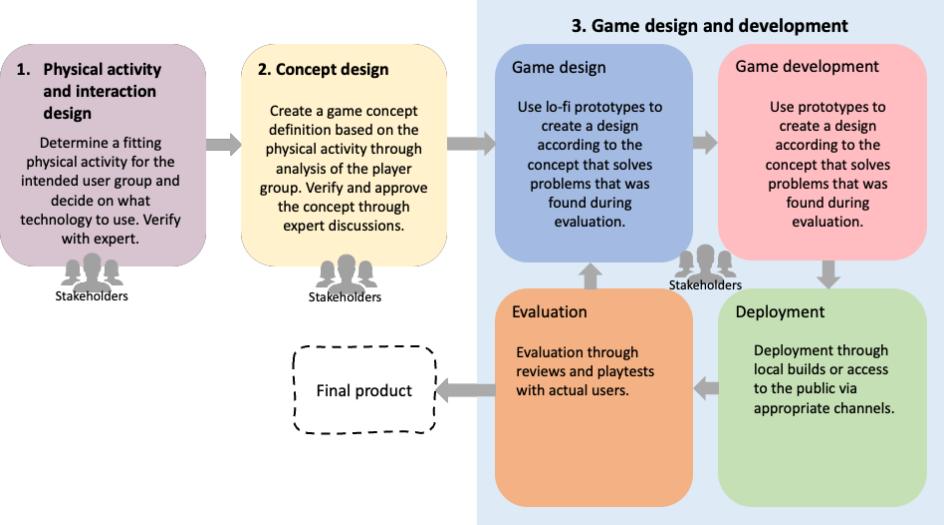
An updated version of the P-III framework established for the design and development of exergames.

The serious game P-III framework [27] needs to be modified in order to be used for the design and development of exergames for health. In Figure 6, we propose an update of the framework adapted for developing exergames that includes three phases: (1) physical activity and interaction design, (2) concept design, and (3) game design and development. The update is inspired by the human-centered design process [32-34] that involves an iterative cycle in four steps: (1) specify the context where the product will be used, (2) specify the user requirements, (3) produce design solutions, and (4) evaluate solutions against these requirements.

### Comparison With Prior Work

We used the serious game P-III framework [27] to design and develop an exergame that need to meet the needs of patients with heart failure [37]. The exergame was developed in 7 iterations.

Since the P-III framework [27] is developed for serious games, in general, it lacks focus on the physical activity and physical interaction design process which is required early in the process of designing exergames. The primary purpose of many exergames is to promote and increase physical activity, and consequently, enhance health [15]. An efficient exergame guarantees that players can reap the desired health advantages. Developers can make sure that the exergame successfully serves the stated health and fitness goals by giving the physical activity component top priority early in the design process and continuously during the development process. Exergames require certain key technical components like motion detection [51], gesture recognition [51,52], and feedback mechanisms [43]. By beginning with physical activity, developers can address these technical issues early in the design process and make sure that the selected technology is in line with the planned player experience. By emphasizing movement and interaction in design, exergames can be made more enjoyable and engaging for players over time by encouraging them to be physically active [43]. Exercise mechanisms can be smoothly incorporated into the exergames by developers if they begin with an emphasis on physical activity. This guarantees that the movements needed for fitness are in line with the goals of the exergame, enhancing the gaming experience.

Evaluations were conducted during each iteration by both the team and users or patients. All concepts and prototypes were reviewed and tested by the interdisciplinary team and target audience. This is in line with the player-centered design approach [27,30,31]. Since the exergame was developed for a smartphone, the evaluations were not solely done on the development computer but also on the intended platforms; Android and iPhone smartphones that support augmented reality. New features were first tested on a desktop and then on the intended platform. By using player simulations to assess the exergame on the desktop with the intended use, development productivity was boosted as testing on the intended platform is typically expensive.

Taking physical activity into account from the beginning enabled the team to handle safety and health issues and take these aspects into consideration during the concept design. The physical interaction is impacted by the platform. Therefore, it is essential that the exergame is continuously deployed on the intended platform to be evaluated so the evaluation is not solely done on the development computer. Using prototypes is good but not sufficient [53] since they serve as the embodiment or rudimentary exploration of concepts and ideas to assess their inherent potential [54]. Interfaces, screen sizes, and input techniques differ throughout systems. Further, prototypes can also be used to explore one area at the expense of another [55]. For example, a technical prototype is made without considering the user experience in order to establish the technical capabilities [55]. Therefore, developers need to evaluate the player experience in the actual setting where the exergame will be played by testing on the target platform. This entails taking into account elements like ergonomics, reactivity, and visibility. Previous research about understanding the relationship between technology and users has shown that deployments are crucial to research investigation [53]. Deployments enable users to use the technology over time in the intimate and disputed situations of daily life which reveals to design research teams the extent to which specific features of the technology are successfully attained [53].

Developing the exergame in an interdisciplinary team was found to be important to ensure that the exergame met the patients’ needs. Stakeholders can be involved in any, potentially multiple, of the following roles [36]: (1) users: stakeholders are treated as users and are involved in the beginning or end of the process, (2) testers: stakeholders test concepts, prototypes, and possible alternatives so the team gets relevant feedback early to improve the game quality and reduces the overall cost of the project, (3) informants: stakeholders act as consultants to the team by sharing ideas during the process, and (4) design partners: stakeholders are involved in contributing to the game design, so prototypes are developed collaboratively.

Having stakeholders with different roles (eg, as domain experts testing the exergame during evaluation, providing information about the patients during concept design, and acting as design partners during the physical activity and interaction design phase) helped the development team to keep the focus on the patient’s needs and to design and develop an exergame that meets these needs. According to Mildner and Mueller [36], constant collaboration with stakeholders helps to keep design efforts on track. As for the P-III framework [27], interdisciplinary teamwork is an important pillar for designing and developing exergames. In our study, we had many iterations with quick turnarounds and feedback from stakeholders, so patients and “healthy” users were included in all phases. As recommended by Pirovano et al [56], users who were not patients were included as they could provide relevant input and were easier to recruit than patients. However, interpretation of evaluation results needed to be made by keeping the target patient group in mind.

### Future Directions

Further research is needed to test the exergame with different patients and to implement and investigate the feasibility and effectiveness of using the exergames by the target group, older adults with heart failure.

### Conclusions

The P-III framework for serious games was applied for the design and development of an exergame for patients with heart failure. A mobile exergame was developed iteratively in 7 iterations with the inclusion of continuous evaluation by the team and with stakeholders. During the development, stakeholders had different roles that helped the development team to keep the focus on the patient’s needs and to design and develop an exergame that met these needs. Based on our case study, we propose an updated version of the P-III framework for exergame design and development: (1) inclusion of separate and thorough prototyping of the physical activity and physical interaction design with high-fidelity prototypes and user testing, and (2) launching the exergame as soon as possible and continuously on the intended platform to enable evaluation and everyday life testing.

## Abbreviations

P-III: player-centered, iterative, interdisciplinary, and integrated
